# Impact of enteral nutrition initiated within 24 h of ECMO on nutritional status and inflammatory response in children

**DOI:** 10.3389/fped.2025.1505935

**Published:** 2025-03-31

**Authors:** Ye Ren, Siwei Lu, Yingfu Chen, Yuelin Sun, Yueqiang Fu, Chengjun Liu, Jing Li, Hongxing Dang

**Affiliations:** ^1^ Department of Pediatric Intensive Care Unit, Ministry of Education Key Laboratory of Child Development and Disorders, Children’s Hospital of Chongqing Medical University, Chongqing, China; ^2^ National Clinical Research Center for Child Health and Disorders, China International Science and Technology Cooperation Base of Child Development and Critical Disorders, Chongqing, China; ^3^ Chongqing Key Laboratory of Child Health and Nutrition, Chongqing, China

**Keywords:** extracorporeal membrane oxygenation, enteral nutrition, nutritional status assessment, inflammation, pediatrics patient

## Abstract

**Objective:**

Malnutrition remains a significant issue in children undergoing ECMO. This study aimed to investigate the effects of initiating enteral nutrition (EN) within 24 h on the adequacy of nutrient intake, nutritional status, anabolic metabolism, and inflammatory markers in children receiving ECMO.

**Methods:**

This was a prospective observational cohort study, including children receiving ECMO therapy at the Children's Hospital of Chongqing Medical University of China from April 2018 to August 2024. Patients were divided into early EN (EEN) and late EN (LEN) groups based on whether effective EN was initiated within 24 h after the start of ECMO. *T*-tests or Mann–Whitney *U* and Chi-square tests were used to compare the clinical characteristics, serum total protein (TP), nutritional intake, serum cholinesterase (CHE), and C-reactive protein (CRP) levels between the two groups. Linear mixed-effects models (LME) were applied to assess the effect of EEN on changes in CRP and CHE levels over time during ECMO.

**Results:**

A total of 47 children were included in this study, with 24 patients (51.1%) successfully receiving EEN. The PRISM3 score was higher in the LEN group (*P* = 0.016). The majority of children in the EEN group had pneumonia or ARDS (*P* < 0.001). The average daily energy and protein intake, as well as their adequacy, was higher in the EEN group compared to the LEN group (*P* < 0.001), although the EEN group experienced more frequent interruptions in EN (*P* < 0.05). Serum TP levels in the EEN group were higher than those in the LEN group during the first 3 days of ECMO (*P* < 0.05). The median CHE levels were higher, and the median CRP levels were lower in the EEN group compared to the LEN group (*P* < 0.05). LME analysis showed a significant interaction effect between EEN and time on CRP and CHE levels (*P* < 0.001).

**Conclusion:**

Successfully initiating EN within 24 h significantly improves the nutritional status of children receiving ECMO, promotes hepatic anabolic metabolism, and reduces inflammatory responses. This study provided new insights and data support for nutritional therapy strategies in children on ECMO.

## 1 Introduction

Enteral nutrition (EN) support is an essential component of the treatment for critically ill patients. Despite EN has been widely promoted in critical care, but the incidence of malnutrition during intensive care remains high due to insufficient attention (1, 2). Particularly in patients receiving extracorporeal membrane oxygenation (ECMO) therapy, inadequate intake of calories and protein during the initial stage is a common and serious issue, partly caused by the effects of extracorporeal circulation and the suppression of gastrointestinal function (3). In addition, ECMO therapy activates a systemic inflammatory response (4), leading to increased catabolism and further exacerbating the consumption of energy and protein. This is particularly concerning in children, as their insufficient protein and energy reserves further elevate the risk of malnutrition, which may even result in organ dysfunction (5, 6). Studies have shown that if the EN intake of children receiving ECMO approaches nutritional targets, survival rates can significantly improve (7).

In 2022, the Extracorporeal Life Support Organization published the first guidelines on nutritional support and assessment for neonates and children receiving ECMO therapy (8), recommending that EN be initiated within 48 h once the child's clinical condition stabilizes. This recommendation aligns with the European Society of Pediatric and Neonatal Intensive Care (ESPNIC) guidelines, which also suggest that EN should be started as early as possible, provided gastrointestinal function allows (9). However, there is currently no consensus on the definition of “early enteral nutrition (EEN),” with the time frame ranging from 6 to 48 h (10, 11). Given the unique characteristics of children, the optimal timing for EN initiation requires further research and discussion.

In the early stages of ECMO therapy, children are typically in a state of high stress, accompanied by a significant inflammatory response. Studies have shown that early initiation of EN may help alleviate the inflammatory response in critically ill patients and improve organ function, particularly gastrointestinal function (10, 12). However, there is a lack of systematic research on the specific timing, clinical outcomes, and particularly the impact on nutritional status and inflammatory response of EEN in children receiving ECMO. Therefore, determining the optimal timing for EN initiation and its clinical benefits during ECMO is crucial for optimizing nutritional support strategies in children on ECMO.

The present study aimed to investigate the impact of initiating EN within 24 h on the adequacy of nutritional intake, nutritional status, liver synthetic function, and inflammatory markers in children undergoing ECMO. We focused on observing changes in serum total protein (TP), cholinesterase (CHE), and C-reactive protein (CRP), with the aim of providing recommendations for EEN in children undergoing ECMO. This study provides clearer guidance and more evidence to support the optimization of nutritional support strategies in clinical practice.

## 2 Material and methods

### 2.1 Study subjects

This prospective observational cohort study was conducted in two Pediatric Intensive Care Units (PICUs) at the Children's Hospital of Chongqing Medical University and the Chongqing Key Laboratory of Child Health and Nutrition in China. The study included data from all children undergoing ECMO between June 2018 and August 2024. The Children's Hospital of Chongqing Medical University is a tertiary pediatric teaching hospital, with its two PICUs designated as China national key clinical specialties. This study did not involve any changes to the treatment plans of the children. All data were anonymized, and the observed indicators did not involve patient privacy. Ethical approval was obtained from the hospital ethics committee, and informed consent was secured from the children and/or their guardians.

### 2.2 Inclusion and exclusion criteria

Inclusion criteria: (1) children receiving V-A mode or V-V mode ECMO therapy; (2) No contraindications to EN; (3) Age ≤ 18 years.

Exclusion criteria: (1) Pre-existing primary gastrointestinal disease leading to gastrointestinal dysfunction before ECMO therapy; (2) Duration of ECMO therapy less than 72 h; (3) Incomplete or unavailable medical records.

### 2.3 Nutritional goals and relevant definitions

The nutritional treatment plan for each child was jointly developed by the attending physicians in the PICU and clinical nutritionists according to clinical protocols. The energy and protein intake targets for each child were calculated based on the Schofield equation. Children were assessed in detail every 4 h, and EN was initiated as soon as no contraindications to EN were confirmed. Feeding tolerance was continuously evaluated after the start of EN. Feeding intolerance was defined as gastric residuals greater than 50% or the occurrence of severe gastrointestinal complications, including necrotizing enterocolitis, gastrointestinal bleeding, stress ulcers, severe abdominal distension, diarrhea, and vomiting. If significant feeding intolerance occurred, EN was suspended and reassessed after 4 h. If EN could not be tolerated for more than 3 days, parenteral nutrition (PN) was supplemented as necessary. EN interruption was defined as the cessation or delay of the EN plan for more than 2 h. Nutritional adequacy was defined as the ratio of actual intake to target intake reaching 30% (13, 14). The definition of “early” is based on the ECMO startup time. EEN was defined as enteral nutrition initiated within 24 h of ECMO initiation, while LEN was defined as initiation beyond 24 h. The PRISM3 score (15) was assessed during the first 24 h of ECMO therapy. Age and weight *z*-scores were calculated using the online tool available at https://reference.medscape.com/guide/medical-calculators.

### 2.4 Observation indicators

① General data: age, weight, gender, length of ICU stay, admission diagnosis, and discharge outcomes; ② Nutritional indicators: time to EN initiation, number of EN interruptions, daily energy and protein intake and adequacy, and serum TP and CHE levels during the first 7 days of ECMO therapy; ③ Inflammatory indicators: for inflammatory markers, if multiple CRP values were obtained on the same day, the median value was used for analysis; ④ ECMO-related indicators: ECMO mode, ECMO flow rate, duration of ECMO, PRISM3 score before ECMO, vasopressor-inotropic score (VIS) before ECMO, use of CRRT, oxygenation index (OI) before ECMO, duration of mechanical ventilation, and other ventilator-related parameters.

### 2.5 Statistical analysis

Data were analyzed using SPSS version 27.0. For continuous variables, normally distributed data were presented as mean ± standard deviation, while non-normally distributed data were described as median (interquartile range). Group comparisons of means and medians were conducted using *t*-tests or Mann–Whitney *U* tests. Categorical variables were described using case counts (percentages) and compared using the chi-square test or Fisher's exact test. To assess the effect of EEN on the temporal changes in CRP and CHE levels during ECMO, a linear mixed-effects model (LME) analysis was performed using the lme4 package in R software. Fixed effects included EEN, time (days), and the interaction between EEN and time. Individual differences among children were modeled as random intercepts to account for baseline variations in CRP and CHE levels. Statistical significance was defined as *P* < 0.05.

## 3 Results

### 3.1 Study flow

**Figure 1:**
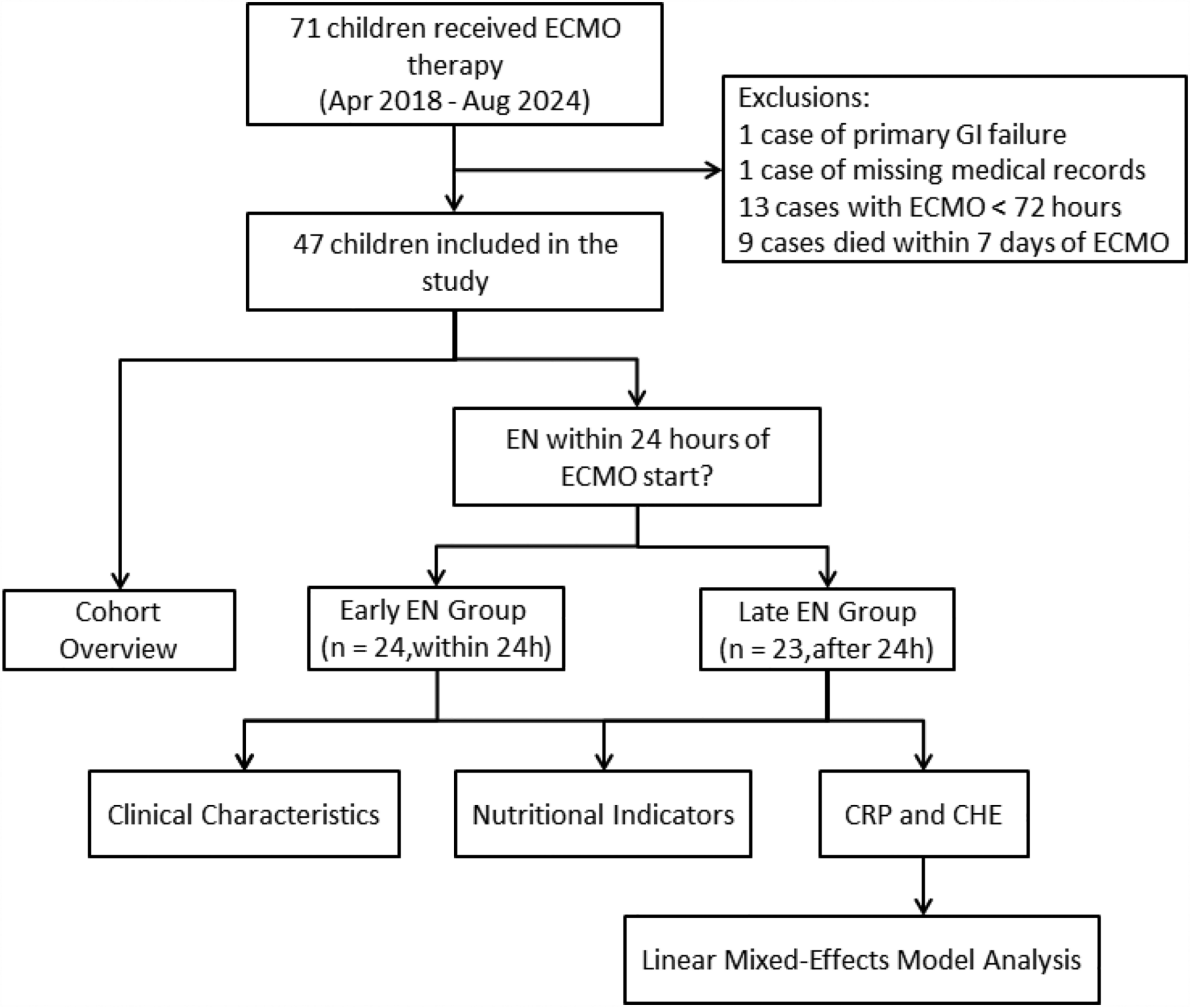
Study flow study flow. ECMO, extracorporeal membrane oxygenation; EN, enteral nutrition; CRP, C-reactive protein; CHE, cholinesterase; GI, gastrointestinal.

During the study period, a total of 71 children received ECMO therapy, with 47 eligible children included in the final analysis. Figure 1 illustrates the study flow and the reasons for excluding certain participants.

### 3.2 Comparison of demographics, clinical characteristics, and outcomes between the two groups

The average time to EN initiation was 53 h after the start of ECMO. The majority of ECMO cases were due to severe pneumonia or ARDS (*P* < 0.001), with myocarditis and congenital heart disease being the other causes. The PRISM3 score in the LEN group was higher than in the EEN group (*P* < 0.05), and 72.3% of the children survived and were discharged. A total of 24 children (51.1%) successfully initiated EEN within 24 h of ECMO start. There were no statistically significant differences in most other relevant clinical characteristics between the EEN and LEN groups (Table 1), and the mechanical ventilation parameters prior to ECMO initiation were also similar between the two groups (Table 2).

**Table 1:** Comparison of demographics, clinical characteristics, and outcomes between the EEN and LEN groups.

Variables	Cohort overview	EEN group (*n* = 24)	LEN group (*n* = 23)	*P*
Weight (Kg)	11.0 (7.2, 22.5)	11.2 (9.2, 22.6)	11.0 (4.9, 22.2)	0.437
Age (M)	24.0 (6.2, 77.5)	23.0 (9.9, 74.7)	24.0 (1.2, 79.0)	0.400
Weight-for-age *Z*-score	−0.69 (−1.38, 0.40)	−0.70 (−1.71, 0.44)	−0.51 (−1.34, 0.11)	0.709
Male, *n* (%)	26 (55.3)	12 (50)	14 (60.9)	0.6485
Time to EN initiation (h)	53 (18.5, 92.5)	18.5 (2, 20.5)	78.5 (28.5, 140.0)	<0.001
ECMO initiation time (h)	74.4 (54.9, 103.5)	77.1 (52.9, 110.8)	70.4 (55.3, 89.5)	0.328
VIS before ECMO	5 (5.0, 15.5)	5.1 (5.0, 10.0)	5 (5.0, 11.8)	0.595
ECMO flow rate (ml/kg/min)	88.0 (74.3, 105.4)	88.7 (73.3, 97.6) 24.7	85.7 (75.9, 108.6)	0.855
V-A ECMO mode, *n* (%)	39 (82.9)	21 (87.5)	18 (78.3)	0.461
Duration of ECMO (h)	111.0 (70.3, 129.5)	88.75 (66.0, 119.2)	114.0 (92.3, 159.0)	0.0721
CRRT, *n* (%)	25 (53.1)	11 (45.8)	14 (60.8)	0.459
PRISM3	16.72 ± 7.28	14.25 ± 6.30	19.30 ± 7.46	0.0156
Length of hospital stay (days)	31.0 (25.0, 50.0)	31.5 (26.0, 49.2)	31.0 (23.5, 50.0)	0.6241
Diagnosis of pneumonia/ARDS, *n* (%)	31 (65.9%)	22 (91.6%)	9 (39.1%)	<0.001
Length of PICU stay (days)	18.0 (14.0, 23.5)	18.0 (14.0, 24.2)	17.0 (12.5, 21.0)	0.4056
OI (Pre-ECMO)	31.0 (26.0, 35.0)	30.5 (24.0, 33.5)	33.0 (26.0, 37.0)	0.262
Survival to discharge, *n* (%)	34 (72.3)	20 (83.3)	14 (60.8)	0.163

EEN refers to EN initiation within 24 h after ECMO start, while LEN refers to EN initiation more than 24 h after ECMO start. ECMO, extracorporeal membrane oxygenation; VIS, vasopressor-inotropic score; V-A mode, veno-arterial mode; CRRT, continuous renal replacement therapy; Time to EN initiation (h); from ECMO initiation to the start of EN; ECMO initiation time (h), from PICU admission to ECMO initiation; OI, oxygenation index. Quantitative data are presented as median (range), and categorical data were presented as case count (percentage).

**Table 2:** Comparison of mechanical ventilation parameters before ECMO between the EEN and LEN groups.

Variables	EEN group (*n* = 24)	LEN group (*n* = 23)	*P*
PIP (cmH_2_O)	33.0 (32.0, 35.0)	37.0 (32.0, 39.0)	0.086
PEEP (cmH_2_O)	9.0 (8.0, 9.0)	9.0 (9.0, 11.0)	0.033
FiO₂ (%)	90.0 (85.0, 95.0)	90.0 (75.0, 97.5)	0.750
MAP (cmH_2_O)	18.0 (17.0, 18.0)	18.0 (18.0, 19.0)	0.074
RR (bpm)	32.0 (29.0, 34.0)	29.0 (28.0, 32.0)	0.085
Duration of mechanical ventilation (h)	264.8 (178.7, 404.4)	277.5 (198.0, 501.2)	0.366

Mechanical ventilation was in volume-controlled mode. EEN: EN initiation within 24 h after ECMO start; LEN: EN initiation more than 24 h after ECMO start. PIP, peak inspiratory pressure; PEEP, positive end expiratory pressure; FiO₂, fraction of inspired oxygen; MAP, mean airway pressure; RR, respiratory Rate; RR, respiratory rate. Quantitative data were presented as median (range).

### 3.3 Comparison of nutritional intake between the two groups

The EN protein and energy intake, as well as adequacy, were significantly higher in the EEN group compared to the LEN group (*P* < 0.001). The calorie and protein intake from PN in the LEN group was slightly higher than in the EEN group, but there was no overall significant difference. There was also no significant difference in additional albumin infusions. None of the children received immuno-enhancing agents, and all children receiving enteral nutrition were fed via nasogastric tubes using an intermittent feeding schedule. However, the frequency of EN interruptions was significantly higher in the EEN group than in the LEN group (*P* < 0.05). A comparison of energy and protein intake from EN and PN, EN interruptions, and albumin infusions during ECMO in both groups was presented in Table 3.

**Table 3:** Comparison of nutritional intake between EEN and LEN groups.

Variables	EEN group (*n* = 24)	LEN group (*n* = 23)	*P*
Average EN energy intake (kcal/kg/d)	19.35 (12.29, 22.24)	9.8 (7.1, 12.9)	<0.001
Average EN protein intake (g/kg/d)	0.53 (0.30, 0.64)	0.19 (006, 0.25)	<0.001
EN energy intake adequacy (%)	33.5 (24.7, 47.3)	11.0 (6.5, 14.5)	<0.001
EN protein intake adequacy (%)	35 (20.0, 43.5)	10.0 (2.0, 14.5)	<0.001
Number of EN interruptions	2.0 (1.0, 3.8)	1.0 (0.0, 2.0)	0.028
Average daily EN interruptions	0.21 (0, 0.65)	0.26 (0, 1.03)	0.248
Average albumin infusion (g/kg/d)	11.5 (2.1, 32.6)	21.8 (5.8, 45.5)	0.115
Average PN energy supply (kcal/kg/d)	0.31 (0, 1.1)	0.71 (0.3, 2.3)	0.127
Average PN amino acid intake (g/kg/d)	19.35 (12.29, 22.24)	9.8 (7.1, 12.9)	<0.001

### 3.4 Comparison of serum TP, CRP, and CHE between the two groups

**Figure 2:**
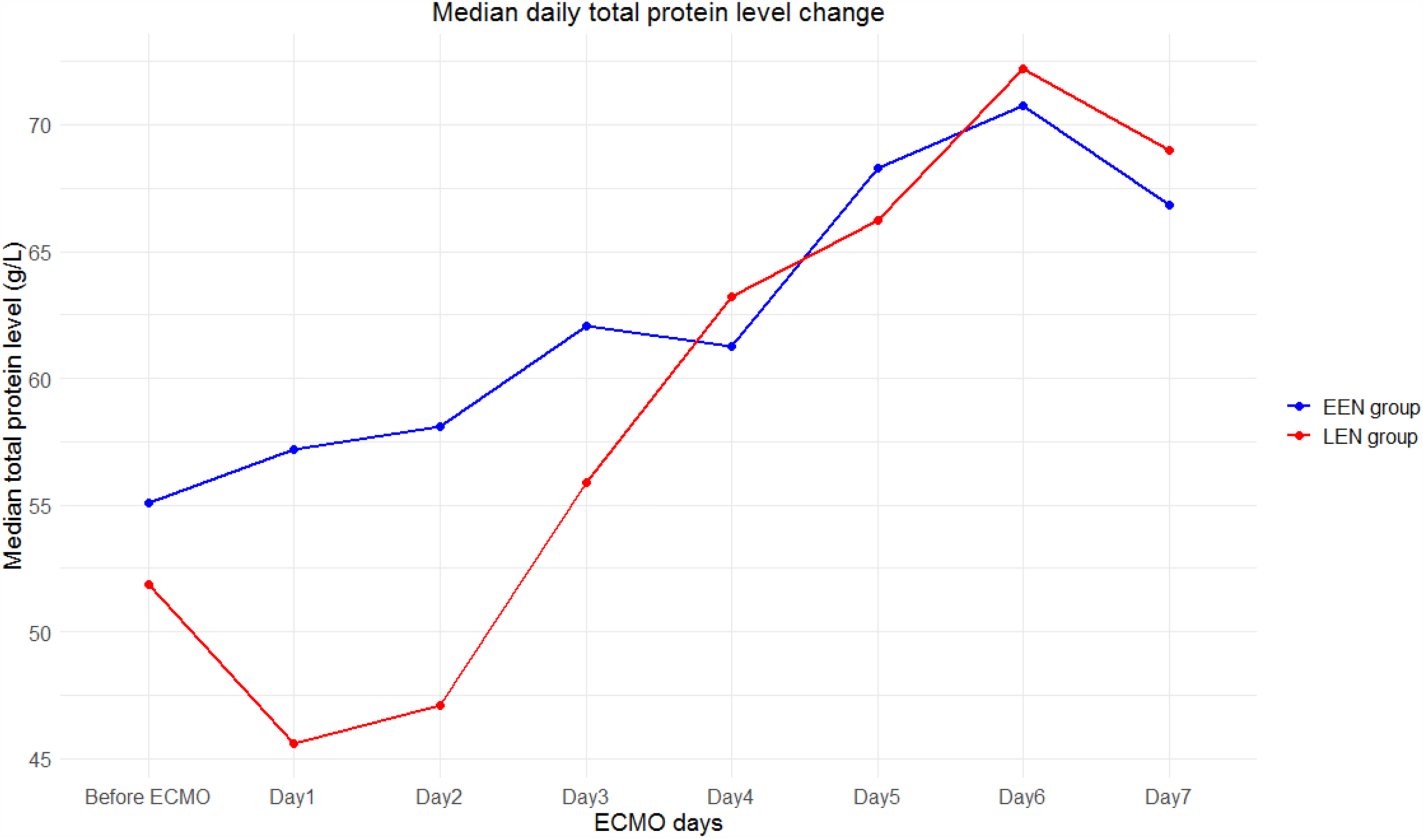
The trend of total protein levels in the two groups during ECMO.

There was no significant difference in serum TP levels between the two groups before ECMO therapy. However, during the first 3 days of ECMO, the TP levels in the EEN group were significantly higher than those in the LEN group (*P* < 0.05). While CRP and CHE levels on the first day of ECMO showed no significant differences between the two groups (*P* > 0.05), the EEN group had a significantly lower average CRP and a higher average CHE compared to the LEN group during the 7 days of ECMO (*P* < 0.05) (Table 4). The trend in serum TP levels during ECMO for both groups was shown in Figure 2.

**Table 4:** Comparison of serum TP, CRP, and CHE between the EEN and LEN groups.

Variables	EEN group (*n* = 24)	LEN group (*n* = 23)	*P*
Serum TP (g/L)			
Before ECMO	53.8 (48.17, 60.97)	50.9 (48.05, 56.05)	0.254
ECMO Day 1	56.7 (51.57, 65.1)	44.7 (39.48, 49.05)	0.008
ECMO Day 3	62 (55.6, 68.1)	52.8 (48.4, 62.9)	0.030
ECMO Day 5	67.3 (65.15, 73.92)	67.3 (61.72, 70.23)	0.712
ECMO Day 7	68.9 (61.05, 73.87)	70.6 (67.15, 71.95)	0.826
CRP on Day 1 (mg/L)	31.5 (17.0, 65.0)	20.0 (13.0, 38.0)	0.125
Average CRP (mg/L)	23.79 (12.27, 30.96)	33.57 (27.29, 46.00)	0.008
CHE on Day 1 (U/L)	3,413.0 ± 1,230.5	3,051.0 ± 1,323.4	0.337
Average CHE (U/L)	4,298.3 ± 886.3	3,347.4 ± 887.3	0.001

TP, serum total protein; CRP, C-reactive protein; CHE, serum cholinesterase.

### 3.5 Analysis of the effect of EEN on CRP and CHE levels

**Figure 3:**
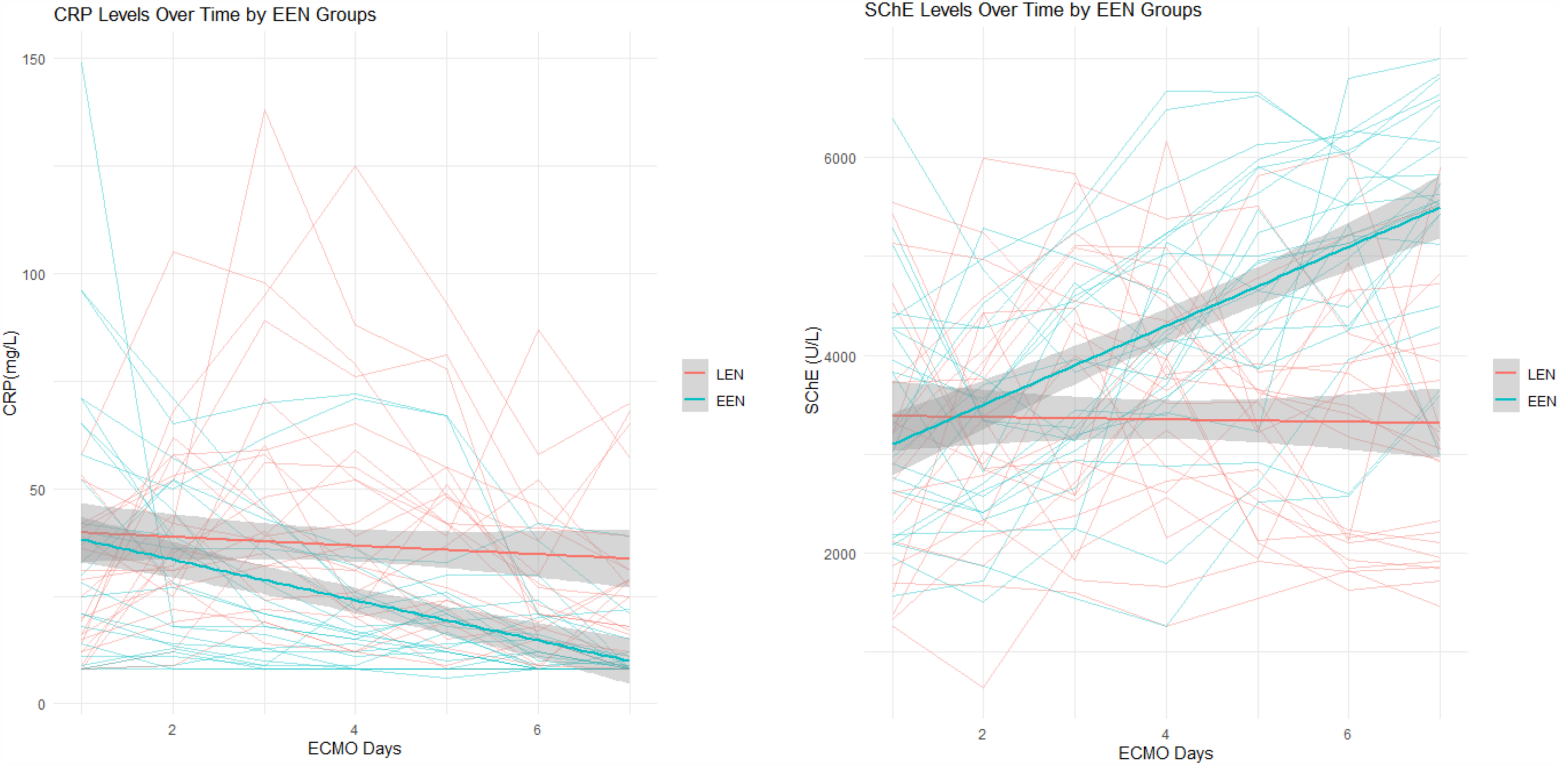
Results of the mixed-effects model: trends in CRP and CHE changes over time.

The analysis of the effect of EEN on CRP and CHE levels using a linear mixed-effects model showed a significant difference in the interaction between EEN and time on both CRP and CHE levels. In the CRP model, the independent effect of EEN on CRP was not significant (*P* = 0.716), but the interaction effect between EEN and time was significant (*P* < 0.001), indicating that CRP levels in children receiving EEN decreased significantly over time, with an additional reduction of 3.69 mg/L per day. In the CHE model, the independent effect of EEN on CHE was significant (*P* = 0.034), with CHE levels in the EEN group being on average 695.34 U/L lower than those in the LEN group. The interaction effect between EEN and time was also significant (*P* < 0.001), showing that CHE levels in the EEN group increased significantly over time, with an increase of 411.47 U/L per day. The independent effect of time on both CRP and CHE was not significant (CRP: *P* = 0.133; CHE: *P* = 0.726), suggesting that time alone had a minimal impact on the changes in both markers. Additionally, PRISM3 was included in the model as a covariate to adjust for baseline disease severity, but did not significantly affect either CRP (*P* = 0.167) or CHE (*P* = 0.933) (Table 5; Figure 3).

**Table 5:** Fixed effect estimates from the linear mixed-effects model for the impact of EEN on CRP and CHE.

Variables	CRP				CHE			
	Estimate	Std. error	*t* value	*P* value	Estimate	Std. error	*t* value	*P* value
Intercept	47.98	6.67	7.19	<0.001	3,422.41	384.57	8.89	<0.001
EEN	2.04	5.82	0.35	0.716	−695.34	326.42	−2.13	0.034
Time (day)	−1.00	0.66	−1.51	0.133	−12.24	34.85	−0.35	0.726
PRISM3	−0.43	0.31	−1.39	0.167	−1.54	18.17	−0.08	0.933
EEN:Time	−3.69	0.93	−3.95	<0.001	411.47	48.77	8.43	<0.001

The table presents the fixed effect estimates from the linear mixed-effects model for CRP and CHE, evaluating the impact of EEN, time, their interaction (EEN:Time), and PRISM3 on CRP and CHE levels. CRP represents C-reactive protein (mg/L), CHE represents cholinesterase (U/L), and PRISM3 represents the pediatric risk of mortality 3 score. EEN is a binary variable indicating whether early enteral nutrition (EEN) was received or not. Time represents the number of observation days (from Day 1 to Day 7). PRISM3 is a covariate used to adjust for baseline disease severity. The EEN: Time interaction effect indicated the differences in the changes of CRP and CHE levels over time between the EEN and LEN groups. A *P*-value of less than 0.05 indicates statistical significance.

## 4 Discussion

Inadequate nutritional intake is common among patients requiring ECMO therapy and is associated with increased ICU mortality (16). The extracorporeal circulation process activates a systemic inflammatory response, which increases energy and protein consumption, further elevating the risk of malnutrition and even leading to organ injury (17). Early nutritional intervention has been shown to significantly benefit the survival of ECMO patients (18). However, research on children is still deficient.

This study analyzed the application of EEN in children undergoing ECMO, revealing the effects of EEN on nutritional intake adequacy and nutritional status, and explored the preliminary impact of EEN on liver synthetic function and the inflammatory response. The results showed that initiating EEN within 24 h after ECMO start significantly improved protein and energy intake and increased serum TP levels. The interaction between EEN and time had a significant effect on increasing CHE and reducing CRP levels, and independent of baseline disease severity. These findings support the positive role of EEN initiation in children undergoing ECMO, highlighting the clinical importance of optimizing nutritional support strategies during ECMO therapy.

Unlike the traditional definition of EEN as EN initiated within 48 h, a key feature of this study was defining EEN as EN initiated within 24 h of ECMO start. The main objective was to evaluate whether further shortening the time to EN initiation in the early phase of ECMO could result in more significant clinical benefits. Children on ECMO are often in an unstable metabolic state, especially during the early stages of treatment when the inflammatory response is most intense. Earlier nutritional intervention may help children manage initial stress more effectively by reducing the inflammatory response and sustaining energy metabolism (6).

Patients receiving ECMO therapy typically enter a state of high metabolic rate and increased protein catabolism, which elevates their nutritional needs (16). In this study, there were no significant differences in the overall clinical characteristics, mechanical ventilation parameters and ECMO therapy processes between the EEN and LEN groups. However, EEN significantly improved the energy and protein intake and intake adequacy in children undergoing ECMO. Therefore, earlier initiation of EN can replenish energy and protein reserves sooner, preventing the further worsening of negative nitrogen balance and thereby helping to avoid a vicious cycle of malnutrition.

Studies have shown that EEN can significantly reduce infection rates and mortality in critically ill patients, while also shortening hospital stays (11, 19). However, the implementation of EEN in clinical practice faces realistic challenges, often leading to feeding interruptions due to gastrointestinal complications and the use of related medications, such as sedatives and vasopressors (20–22). This is consistent with the findings of this ECMO study, where the number of EN interruptions in the EEN group was significantly higher than in the LEN group. This may be due to gastrointestinal hemodynamic changes in the early stages of ECMO initiation, where some children's gastrointestinal function may not fully tolerate EN. Nevertheless, despite the increased number of interruptions, the total energy and protein intake in the EEN group remained still significantly higher than in the LEN group, demonstrating the overall advantages of EEN in ECMO.

In cases where EN cannot be tolerated, moderately increasing the proportion of PN may be a practical clinical solution (23). In this study, some children undergoing ECMO received PN. Although PN can partially meet the energy needs of children, it has clear limitations, particularly in promoting gastrointestinal function recovery and reducing infection risks (13). This suggests that the EN strategy must be flexibly adjusted according to the individual condition of each child, combining PN when necessary to compensate for nutritional deficiencies caused by interruptions. Additionally, the synergistic effects of EN and PN may vary among children, and future studies are needed to further explore the optimal nutritional management strategies to effectively balance feeding interruptions and nutritional goals during the early adaptation phase.

This study showed that serum TP levels in the EEN group were significantly higher than those in the LEN group within the first 3 days of ECMO, indicating that EN more effectively maintained the nutritional status of children during the early stage. Serum TP levels reflect the overall nutritional status of the body, particularly the balance between protein synthesis and consumption. During ECMO, children are typically in a hypercatabolic state, which significantly increases protein requirements. The timely initiation of EEN provides the necessary nutritional substrates to promote protein synthesis and maintain serum TP levels. However, after the 4–5th day of ECMO, the differences in TP levels between the two groups became insignificant, suggesting that the nutritional status of the LEN group also improved as treatment progressed. This improvement may be related to the supplementation of PN and comprehensive clinical interventions in the later stages. Nevertheless, the early advantage of the EEN group in achieving target TP levels more rapidly is still noteworthy, as early nutritional adequacy is crucial for long-term clinical outcomes.

There was no significant difference in CRP levels between the two groups on the first day of ECMO. However, the average CRP levels thereafter were significantly lower in the EEN group compared to the LEN group. This study suggests that early EEN may play a positive role in controlling the inflammatory response in children undergoing ECMO. CRP is a sensitive marker reflecting inflammation and infection in the body, with lower CRP levels generally indicating better inflammation control and prognosis (23). Higher CRP levels are also associated with poorer nutritional status in critically ill patients (24). Previous studies have shown that EN has potential advantages in suppressing inflammation, particularly in critically ill patients, where EEN can notably reduce the risk of infection (25). By providing EEN support, EEN helps maintain intestinal barrier function, reduce bacterial translocation, and mitigate the systemic inflammatory response caused by ECMO or cardiopulmonary failure.

Although there was no significant difference in CHE levels between the EEN and LEN groups on the first day of ECMO, the average CHE levels in the EEN group were significantly higher than those in the LEN group thereafter. This further supports the notion that early nutritional intervention improves the overall metabolic state. CHE is an important indicator of liver function and protein synthesis capacity, and it is associated with mortality in critically ill children (26). An increase in CHE levels usually suggests good liver function and the recovery of anabolic processes in the body. In this study, EEN was shown to help maintain liver function and protein synthesis capacity. This is consistent with existing research, which indicates that malnutrition often leads to a decline in liver synthetic function, while early nutritional intervention can prevent or even reverse this process to some extent (27).

We conducted an in-depth analysis of the effect of EEN on CRP and CHE levels in children undergoing ECMO using a linear mixed-effects model. The results demonstrated that EEN significantly influenced the dynamic changes of these two physiological indicators, particularly in the interaction effects over time, and notably, this effect was independent of disease severity.

In the CRP model, although the independent effect of either EEN or time was not significant, but the significant interaction effect has important clinical implications. This indicates that changes in CRP levels depend more on the combined effect of EEN and time, rather than the independent influence of either time or EEN alone. As a marker of the inflammatory response, the significant decline in CRP levels over time suggests that EEN may reduce the body's inflammatory response and exert immunomodulatory effects through a cumulative effect over time.

In the CHE model, EEN not only had a significant independent effect on CHE levels, but the interaction effect between EEN and time was also significant. CHE levels in the EEN group showed a daily increase of 411.47 U/L, and the overall levels were significantly higher than those in the LEN group. Early and adequate nutritional intake is crucial for maintaining anabolic processes and liver function in patients. As a comprehensive biomarker of malnutrition, the increase in CHE levels reflects the important role of EEN in promoting anabolic metabolism and improving liver function (27). EN helps maintain the integrity of the intestinal mucosa, reducing enterogenic infections, and thereby lessening the metabolic burden on the liver. This cumulative effect over time contributes to the sustained improvement in nutritional status and liver synthetic function.

Another finding of this study is that, although the EEN group showed better outcomes than the LEN group in key clinical indicators such as length of hospital stay, length of PICU stay, duration of mechanical ventilation, ECMO duration, and survival to discharge, but the statistical differences were not significant. This may be due to the limitations in sample size, or it may suggest that the long-term impact of EEN on children's prognosis is more complex. Nutritional factors may be only one aspect influencing prognosis, and an initial EN deficiency may not directly lead to a worse final outcome. The most critical factors affecting prognosis are still likely related to the nature of the underlying disease itself. If EN can be gradually restored in the later stages of ECMO therapy, and supplemented by PN, the impact on survival rates may be limited. However, even if these children survive to discharge, they may face longer recovery periods or other long-term complications. Nevertheless, this study provides new insights and data to support future research and clinical practice.

This study has certain limitations. First, the number of children receiving ECMO therapy is inherently small, although the study spanned 6 years, the sample size remained limited, which may restrict the generalizability of the results. Second, due to the lack of long-term follow-up data, the impact of EEN on long-term prognosis could not be assessed. Third, although this study explored the effects of EEN on nutritional status and inflammatory response, and the major covariates between the two groups were generally balanced, other potential confounding factors may have influenced the results. In addition, due to the lack of detailed pre-ECMO nutrition data and possible differences in the metabolic state at ECMO initiation, the causal inference regarding the relationship between EEN and inflammatory as well as metabolic responses might be affected. Future studies should consider further refined subgroup analyses to address these issues.

## Data Availability

The datasets presented in this study can be found in online repositories. The names of the repository/repositories and accession number(s) can be found below: Dang, Hongxing (2024), “Enteral Nutrition on Nutritional Status and Inflammatory Response in Pediatric ECMO”, Mendeley Data, V1, doi: 10.17632/y2xcs6gxt7.1.
